# Draft genome sequences for the obligate bacterial predators *Bacteriovorax spp.* of four phylogenetic clusters

**DOI:** 10.1186/1944-3277-10-11

**Published:** 2015-03-24

**Authors:** Huan Chen, Lauren M Brinkac, Pamela Mishra, Nan Li, Despoina S Lymperopoulou, Tamar L Dickerson, Nadine Gordon-Bradley, Henry N Williams, Jonathan H Badger

**Affiliations:** 1 Florida A&M University, Tallahassee, USA; 2 National High Magnetic Field Laboratory, Florida State University, Tallahassee, FL 32310-4005, USA; 3 J Craig Venter Institute, Rockville, MD 20850, USA; 4 J Craig Venter Institute, La Jolla, CA 92037, USA

**Keywords:** Predatory bacteria, *Bdellovibrio* and like organisms, *Bacteriovorax*, Marine, Gram-negative, Motile

## Abstract

*Bacteriovorax* is the halophilic genus of the obligate bacterial predators, *Bdellovibrio* and like organisms. The predators are known for their unique biphasic life style in which they search for and attack their prey in the free living phase; penetrate, grow, multiply and lyse the prey in the intraperiplasmic phase. *Bacteriovorax* isolates representing four phylogenetic clusters were selected for genomic sequencing. Only one type strain genome has been published so far from the genus *Bacteriovorax*. We report the genomes from non-type strains isolated from aquatic environments. Here we describe and compare the genomic features of the four strains, together with the classification and annotation.

## Introduction

As a member of the highly diverse *Deltaproteobacteria* class, the obligate bacterial predators *Bdellovibrio* and like organisms possess unique ecological features that are worth exploring. They are the only known predatory bacteria that exhibit a life cycle alternating between an extracellular free-living phase and an intraperiplasmic phase and are capable of invading the periplasmic space of prey cells, resulting in the lysis of the prey and release of new progeny [1]. Based on their small size, about 1/5th that of a typical bacterium cell, BALOs have been called “the world’s smallest hunters”. Nevertheless, their genomes are larger than expected, more than 3.98 Mb in *Bdellovibrio. bacteriovorus* Tiberius [2], 3.78 Mb in *B. bacteriovorus* HD100 [1] and 3.44 Mb in *Bacteriovorax marinus* SJ [3]. Despite the uniqueness [4], and increasing understanding, of the potential of these organisms in various applications [5-7], their phylogeny and unique predatory features are only beginning to be understood.

Systematics has played a most important role in advancing the study of the BALOs. Based on systematic genomic molecular techniques, the original BALO genus, *Bdellovibrio*, has been subdivided into four genera: *Bdellovibrio**,**Bacteriolyticum**,**Peredibacter**, and**Bacteriovorax*[8-10]*.* Being an exclusive saltwater genus, *Bacteriovorax* is distinct from the freshwater/terrestrial members of BALOs in many ways. It is ubiquitous in salt-water environments [10], requires at least 0.5% NaCl for growth, prefers saltwater prey [11], thrive at a lower temperature range [12] and has a lower % GC ratio of ca. 37% [13] compared to the 50.65% of the freshwater *Bdellovibrio bacteriovorus* HD100. Currently, *Bacteriovorax marinus* SJ is the only strain from the genus *Bacteriovorax* of which the complete genome has been sequenced and reported.

To date, variations in the 16S rRNA sequences have yielded approximately eight *Bacteriovorax* clusters or OTUs. The previously sequenced *Bacteriovorax marinus* SJ^T^ is one of the representatives that belong to phylogenetic Cluster III. This classification scheme has enabled for the first time the detection of specific *Bacteriovorax* strains in environmental/ecological studies. The validity of using the 16S rRNA gene was tested by comparison with the *rpoB* gene [10]. The results of recent studies monitoring the activities and distribution of specific phylogenetic clusters have yielded new discoveries on the distribution, predation patterns, prey preferences, and ecology of this bacterial predator [14-16].

Here we present a description of the draft genomes of *Bacteriovorax* isolates of four phylogenetic clusters isolated from estuarine systems, together with the description of the genomic sequencing and annotation.

## Organism information

**Figure 1:**
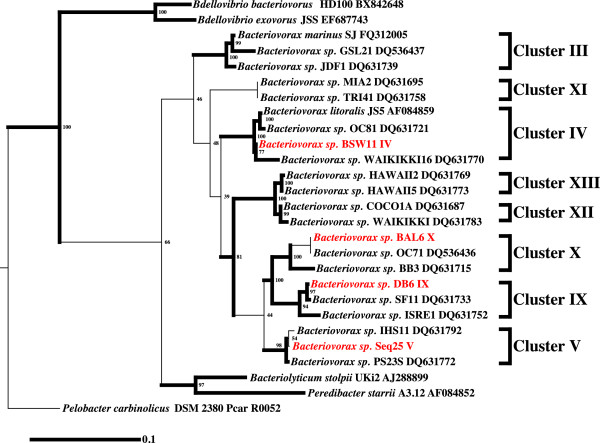
**Phylogenetic tree highlighting (red) the position of four newly sequenced *Bacteriovorax *strains relative to the type strains within the family *Bdellovibrionaceae *and two non-type strains of each *Bacteriovorax *phylogenetic clusters.** The tree was constructed using 16S rRNA gene sequences aligned by the RDP aligner, and was inferred using RaxML 7.25 [17] with the GTRGAMMA model of sequence evolution. The strains and their corresponding GenBank accession numbers for 16S rRNA genes were (type = T): *Bacteriovorax sp.* BSW11_IV; *Bacteriovorax sp.* SEQ25_V; *Bacteriovorax sp.* DB6_IX; *Bacteriovorax sp.* BAL6_X, *Bdellovibrio bacteriovorus* HD100^T^ (BX842648); *Bacteriolyticum stolpii* UKi2^T^ (AJ288899); *Bacteriovorax marinus* SJ^T^ (FQ312005); *Peredibacter starrii* A3.12^T^ (AF084852); *Bx litoralis* JS5^T^ (AF084859); *Bdellovibrio exovorus* JSS^T^ (EF687743); *Bx* sp. BB3 (DQ631715); *Bx* sp. OC71 (DQ536436); *Bacteriovorax* sp. PS23S (DQ631772); *Bx* sp. IHS11 (DQ631792); *Bacteriovorax* sp. SF11 (DQ631733); *Bacteriovorax* sp. ISRE1 (DQ631752); *Bacteriovorax* sp. GSL21 (DQ536437); *Bacteriovorax* sp. JDF1 (DQ631739); *Bacteriovorax* sp. MIA2 (DQ631695); *Bacteriovorax* sp. TRI41 (DQ631758); *Bx sp.* COCO1A (DQ631687); *Bacteriovorax sp.* WAIKIKKI (DQ631783); *Bx sp*. OC81 (DQ631721); *Bacteriovorax sp.* WAIKIKKI16 (DQ631770); *Bacteriovorax sp.* HAWAII2 (DQ631769); *Bx sp.* HAWAII5 (DQ631773). *Deltaproteobacterium*, *Pelobacter carbinolicus* DSM2380 (CP000142), was used as an out-group. The numbers along the branches reflect the proportion of times the groups cluster together based on 100 bootstrapped replicates. Thick branches represent those with greater than 75% bootstrap support. Phylogenetic clusters of *Bacteriovorax* based on 96.5% or greater 16S rRNA gene sequence similarity are denoted by brackets on the right of the tree. Clusters were numbered consistently with previous reports [9,10,18].

A 16S rRNA phylogenetic tree was constructed showing the phylogenetic neighborhood of the four newly sequenced *Bacteriovorax* strains within the family of *Bdellovibrionaceae* (Figure 1). As expected, *Bacteriovorax**sp.* strain BSW11_IV was grouped together with cluster IV, strain SEQ25 _V with cluster V, Strain DB6_IX with Cluster IX and lastly strain BAL6_X with cluster X.

**Figure 2:**
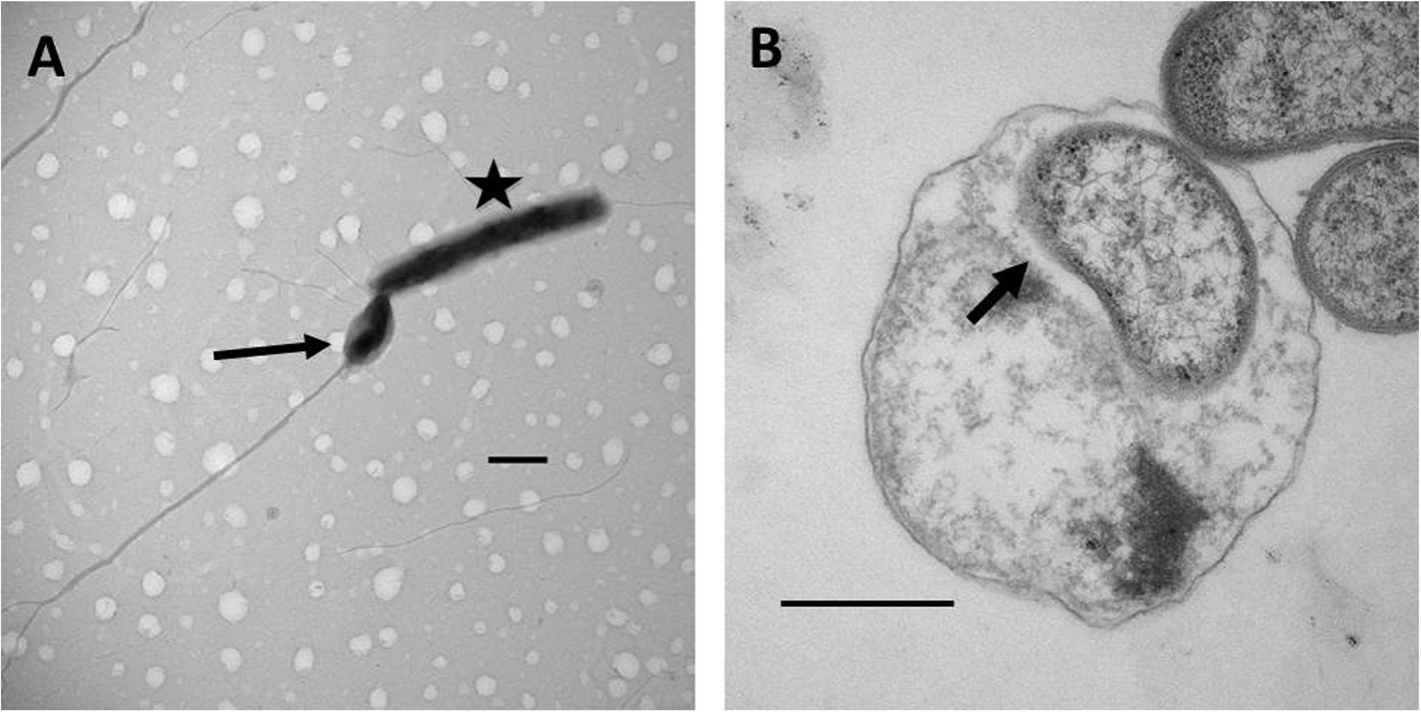
**Electron micrographs showing (A) *Bx sp. *DB6_IX *(arrow) *attached to the polar end of the prey cell *V. vulnificus *(Star)*; *(B) thin sections of bdelloplast, the post-BALO infection structure with the predator (arrow) residing inside the prey cell.** Scale Bar represents 500 nm.

General features of *Bacteriovorax**spp.* are summarized in Table 1. Individual features of *Bacteriovorax* isolates have not been sufficiently explored and are largely unknown. Micrographs generated by both transmission electron microscopy and scanning microscopy (Figure 2) suggest that *Bacteriovorax**spp.* employ similar predation strategies as other BALO members to attack and reside in the periplasic space of its prey.

**Table 1:** **Classification and general features of *Bacteriovorax *** **strains according to the MIGS recommendations **[19]

**MIGS ID**	**Property**	**Term**		**Evidence code**^**a**^
	Current classification	Domain	*Bacteria*	TAS [20]
		Phylum	*Proteobacteria*	TAS [21]
		Class	*Deltaproteobacteria*	TAS [22,23]
		Order	*Bdellovibrionales*	TAS [24]
		Family	*Bacteriovoracaceae*	TAS [25]
		Genus	*Bacteriovorax*	TAS [3]
		Species	Cluster IV, Cluster V, Cluster IX, Cluster X	TAS [18]
		Strains:	BSW11_IV, SEQ25_V, DB6_IX, BAL6_X	IDA
	Gram stain	Negative		TAS [26]
	Cell shape	comma-shaped, 0.35-1.2 μm		TAS [26]
	Motility	motile (one single, polar, sheathed flagellum)		TAS [26]
	Sporulation	Non-sporulating		NAS
	Temperature range	10-35°C		TAS [13]
	Optimum temperature	15-30°C		TAS [3]
	Carbon source	Peptides, proteins		TAS [13]
	Energy source	Chemo-organotroph		TAS [13]
	Terminal electron receptor	Unknown		IDA
MIGS-6	Habitat	marine, estuarine		TAS [18]
MIGS-6.3	Salinity	>0.5%		TAS [3]
MIGS-22	Oxygen	Aerobic		NAS
MIGS-15	Biotic relationship	free living/ parasitic		TAS [3]
MIGS-14	Pathogenicity	Not reported		TAS [3]
MIGS-4	Geographic location	Breton Sound, LA (BSW11_IV);		IDA
		Barataria Bay, LA (SEQ25_V);		
		Apalachicola Bay, FL (DB6_IX, BAL6_X)		
MIGS-5	Sample collection time	April, 2011 (BSW11_IV);		IDA
		June, 2011 (SEQ25_V);		
		October, 2010 (DB6_IX, , BAL6_X)		
MIGS-4.1 MIGS-4.2	Latitude – Longitude	29.63 -89.66 (BSW11_IV);		IDA, TAS [14]
		29.38 -89.98 (SEQ25_V);		
		29.67 -85.09 (DB6_IX, BAL6_X)		
MIGS-4.3	Depth	not reported (BSW11_IV, SEQ25_V);		NAS,TAS [14]
		1.74 m (DB6_IX, BAL6_X)		
MIGS-4.4	Altitude	not reported		IDA

^a^Evidence codes - IDA: Inferred from Direct Assay; TAS: Traceable Author Statement (i.e., a direct report exists in the literature); NAS: Non-traceable Author Statement (i.e., not directly observed for the living, isolated sample, but based on a generally accepted property for the species, or anecdotal evidence). These evidence codes are from the Gene Ontology project [27].

## Genome sequencing information

### Genome project history

The four genomes were selected for sequencing on the basis of their phylogenetic position and isolation source. Low salt *Bacteriovorax**sp.* BSW11_IV was isolated from Breton Sound, Louisiana (salinity 0.6 ppt; Temperature 26.4°C) and SEQ25_V was obtained from water samples of Barataria Bay, Louisiana (salinity 5.2 ppt; Temperature 19.2°C). High salt DB6_IX (Salinity 32.4 ppt; Temperature 24.1°C) and BAL6_ X (Salinity 30.9 ppt; Temperature 25.2°C) were obtained from Apalachicola Bay, Florida. The genome sequences were deposited in GenBank. Sequencing and annotation were performed at the J. Craig Venter Institute. Table 2 presents the project information and its association with MIGS version 2.0 compliance [19].

**Table 2:** Genome sequencing project information

**MIGS ID**	**Property**	**BSW11_IV**	**SEQ25_V**	**DB6_IX**	**BAL6_X**
MIGS-31	Finishing quality	improved-high-quality draft	improved-high-quality draft	improved-high-quality draft	improved-high-quality draft
MIGS-28	Libraries used	3 KB 454 PE, 327 bp avg. insert Illumina fragment	3 KB 454 PE, 335 bp avg. insert Illumina fragment	3 KB 454 PE, 346 bp avg. insert Illumina fragment	3 KB 454 PE, 316 bp avg. insert Illumina fragment
IGS-29	Sequencing platforms	Illumina GAII, 454 GS FLX Titanium	Illumina GAII, 454 GS FLX Titanium	Illumina GAII, 454 GS FLX Titanium	Illumina GAII, 454 GS FLX Titanium
MIGS-31.2	Fold coverage	700× hybrid coverage	85× hybrid coverage	583× hybrid coverage	81× hybrid coverage
MIGS-30	Assemblers	Newbler 2.6	CLC 5.0	CA 7.0	CA 7.0
MIGS-32	Gene calling method	Glimmer 3.02	Glimmer 3.02	Glimmer 3.02	Glimmer 3.02
	Genome Database release	August 16, 2013	August 16, 2013	August 16, 2013	August 5, 2013
	Genbank ID	PRJNA210325	PRJNA210326	PRJNA210327	PRJNA210328
	Genbank Date of Release	August 16, 2013	August 16, 2013	August 16, 2013	August 5, 2013
	GOLD ID	Gi0051698	Gi19265	Gi0051699	G0i005170
MIGS-13	Project relevance	Environment	Environment	Environment	Environment

### Growth conditions and DNA isolation

*Bacteriovorax* cultures were grown separately in 70% artificial sea water (ASW) (Instant Ocean, Aquarium Systems, Inc., Mentor, Ohio) (pH 8, salinity 22 ppt.) amended with prey, *Vibrio. vulnificus* CMCP6 (for *Bx sp.*BSW11_IV and SEQ25_V), or *V. parahaemolyticus*RIMD 2210633 (for *Bacteriovorax**sp.* DB6_IX and BAL6_X). The genomes of both prey bacteria have been sequenced previously [28,29]. When cultures became clear (2–3 days after inoculation of the prey), which indicated the majority of the prey cells were lysed by the predators, 300 ml suspensions were filtered consecutively through 0.45 and 0.22 μm sterile syringe filters (Corning, NY, USA) to remove any remaining prey. Filtrates containing high concentrations of *Bacteriovorax* cells (ca. 4 × 10^8^ PFU ml^−1^) were centrifuged at 27,485 × g for 20 min at 4°C. The pellets were then re-suspended in 1 ml of ASW respectively. To test that the concentrated *Bacteriovorax* suspensions were free of prey cell contamination, aliquots of 0.1 ml of the filtrate were spread-plated onto LB agar and incubated at 37°C for two days.

Subsequently, total DNA from the cell pellets were extracted using the QIAGEN Kit (QIAamp DNA Mini Kit), according to the manufacturer’s protocol. The concentration and purity of DNA was measured by a NanoDrop Spectrophotometer (ND 1000, Thermo Fisher Scientific, DE). To reconfirm the phylotype of the isolations, the DNA was PCR amplified using *Bacteriovorax* specific primers, Bac-676 F (5′-ATT TCG CAT GTA GGG GTA-3′) and Bac-1442R (5′-GCC ACG GCT TCA GGT AAG-3′) [30] by puReTaq Ready-To-Go PCR Beads (GE Healthcare Bio-Sciences). PCR products were purified with the QIAquick PCR-Purification Kit (QIAGEN) and sequenced with Bac-676 F primer at the DNA Sequencing Laboratory at Florida State University. 16S DNA sequences were analyzed with the Basic Local Alignment Search Tool (BLAST) server from the National Center of Biotechnology Information [31].

### Genome sequencing and assembly

Genome sequencing of the four *Bacteriovorax* isolates was conducted at the J. Craig Venter Institute employing a combination of Illumina and 454 sequencing platforms. The 454 data consisted of a half plate of 454 FLX per genome from 3 KB mate paired libraries. The Illumina data consisted of one-quarter lane of 2 × 100 bp Illumina HiSeq data per genome. On average, 300,000 454 reads (average length trimmed 300 bp) and 10 million Illumina sequences (average length trimmed 100 bp) were generated per genome. To incorporate a hybrid assembly using both 454 and Illumina sequence libraries, one million reads were randomly sampled (with their mates) from the Illumina library using Celera [32], which was sufficient to provide high coverage in the initial assemblies.

## Genome annotation

Genes were identified using GLIMMER3 [33] as part of the JCVI prokaryotic annotation pipeline followed by manual curation using the Manatee annotation-editing platform. The JCVI automated pipeline incorporates HMM3[34] searches against Pfam [35] and TIGRFAMs [36] and BLASTP against UniProt [37], JCVI’s database of experimentally characterized proteins CharProt DB [38], and PIR [39].

## Genome properties

The *Bacteriovorax**sp.* BSW11*_* IV draft genome contains 3,650,096 bp with a GC content of 37%. The hybrid assembly was scanned for contamination using BlastP and the appropriate contigs were filtered out. The final assembly comprised of 3 scafolds, 30 RNAs and 3457 CDS. For the CDSs, 2591 (75%) proteins had a BLASTP hit with an e-value of 1e-9 or better to *Bacteriovorax marinus* SJ, and an additional 151 (4%) CDSs had a hit within the genus *Bdellovibrio**.*

The *Bacteriovorax**sp.* SEQ25_V draft genome contains 3,450,786 bp with a GC content of 37%. The sequences were assembled into 29 contigs comprised of 35 RNAs and 3,292 CDSs. Among the CDSs, 2,456 (75%) of proteins had a BLASTP hit with an e-value of 1e-9 or better to *Bacteriovorax marinus**SJ*, and an additional 131 (4%) CDSs had a hit within the genus *Bdellovibrio**.*

The *Bacteriovorax**sp. DB6_*IX draft genome contains 2,969,235 bp with a GC content of 38%. Sequences were assembled into 10 scaffolds with 30 RNAs and 3192 CDSs. Among the CDSs 2,253 (71%) proteins had a BLASTP hit with an e-value of 1e-9 or better to *Bx marinus SJ*, and an additional 97 (3%) CDSs had a hit within the genus *Bdellovibrio**.*

The *Bacteriovorax**sp. BAL6_* X draft genome contains 3,233,679 bp with a GC content of 36%. The reads were assembled into 9 contigs with 37 RNAs and 3,065 CDSs. Among the CDSs, 2,298 (72%) proteins had a BLASTP hit with an e-value of 1e-9 or better to *Bacteriovorax marinus**SJ*, and an additional 92 (3%) CDSs had a hit within the genus *Bdellovibrio**.*

It is noteworthy to point out that three phage tail fiber proteins were identified within the *Bx* sp. BSW11_IV genome but were absent from all the other BALO genome including the completed *Bacteriovorax marinus* SJ and *Bdellovibrio bacteriovorus* HD100 genomes. A staphylococcal phi-Mu50B-like prophage element was present in both SJ and HD 100 genomes but was not found in the genomes of the four newly sequenced *Bacteriovorax* isolates. The properties and the statistics of the genome are summarized in Tables 3, 4 and 5 and (Additional file 1: Table S1).

**Table 3:** Summary of genomes

**Label**	**Size (Mb)**	**Topology**	**INSDC identifier**
BSW11_IV	3.65	Circular	PRJNA210325
SEQ25_V	3.45	Circular	PRJNA210326
DB6_IX	2.97	Circular	PRJNA210327
BAL6_X	3.23	Circular	PRJNA210328

**Table 4:** Nucleotide content and gene count levels of the genome

	**BSW11_IV**		**SEQ25_V**		**DB6_IX**		**BAL6_X**	
	**Value**	**% of total**^**a**^	**Value**	**% of total**^**a**^	**Value**	**% of total**^**a**^	**Value**	**% of total**^**a**^
Genome size (bp)	3,650,096	100.00%	3,450,786	100.00%	2,969,235	100.00%	3,233,679	100.00%
G + C content (bp)	1,347,908	36.93%	1,243,844	36.05%	1,117,420	37.63%	1,179,198	36.47%
Total genes	3,487	100.00%	3,327	100.00%	3,222	100.00%	3,102	100.00%
RNA genes	30	0.86%	35	1.05%	30	0.93%	37	1.19%
Protein-coding genes	3,457	99.14%	3,292	98.95%	3,192	99.07%	3,065	98.81%
Proteins assigned to COGs	2,144	62.02%	2,045	62.12%	1,911	59.87%	1,815	59.22%
Proteins with transmembrane helices	708	20.48%	650	19.74%	578	18.11%	661	21.57%

^a)^The total is based on either the size of the genome in base pairs or the total number of protein coding genes in the annotated genome.

**Table 5:** Number of genes associated with the 25 general COG functional categories

**Code**	**BSW11_IV**		**SEQ25_V**		**DB6_IX**		**BAL6_X**		
	**Value**	**% of total**^**a**^	**Value**	**% of total**	**Value**	**% of total**	**Value**	**% of total**	**Description**
J	166	4.80	167	5.07	137	4.29	163	5.31	Translation
A	0	0.00	0	0.00	0	0.00	0	0.00	RNA processing and modification
K	126	3.64	121	3.67	109	3.41	108	3.52	Transcription
L	115	3.32	103	3.12	100	3.13	112	3.65	Replication and repair
B	1	0.03	1	0.03	2	0.06	1	0.03	Chromatin structure and dynamics
D	24	0.69	27	0.82	23	0.72	29	0.94	Cell cycle control and mitosis
Y	0	0.00	0	0.00	0	0.00	0	0.00	Nuclear structure
V	45	1.3	34	1.03	31	0.97	40	1.30	Defense mechanisms
T	229	6.62	205	6.22	219	6.86	126	4.11	Signal transduction mechanisms
M	155	4.48	161	4.89	129	4.04	140	4.56	Cell wall/membrane/biogenesis
N	95	2.74	82	2.49	68	2.13	71	2.31	Cell motility
Z	2	0.06	3	0.09	2	0.06	3	0.10	Cytoskeleton
W	21	0.61	22	0.67	16	0.50	19	0.62	Extracellular structures
U	43	1.42	43	1.31	38	1.19	44	1.43	Intracellular trafficking and secretion
O	125	3.61	118	3.58	97	3.03	118	3.84	Posttranslational modification, protein turnover, chaperones
C	117	3.38	123	3.73	106	3.32	112	3.65	Energy production and conversion
G	72	2.08	80	2.43	67	2.09	53	1.72	Carbohydrate transport and metabolism
E	192	5.55	166	5.04	166	5.20	138	4.50	Amino Acid transport and metabolism
F	53	1.53s	51	1.54	65	2.03	49	1.59	Nucleotide transport and metabolism
H	76	2.19	83	2.52	72	2.25	73	2.38	Coenzyme transport and metabolism
I	108	3.12	91	2.76	104	3.25	96	3.13	Lipid transport and metabolism
P	90	2.60	84	2.55	88	2.75	87	2.83	Inorganic ion transport and metabolism
Q	54	1.56	60	1.82	69	2.16	48	1.56	Secondary metabolites biosynthesis, transport and catabolism
R	308	8.90	296	8.99	274	8.58	262	8.54	General Functional Prediction only
S	156	4.51	150	4.55	124	3.88	142	4.63	Function Unknown
-	1372	39.68	1293	39.27	1355	42.44	1244	40.58	Not in COG

^a^The total is based on the total number of protein coding genes in the annotated genome.

## Insights from the genome sequences

### Genome Comparisons between BALO Members

Crossman et al., [40] reported that the genomic sequences of *Bacteriovorax marinus* SJ were unique with about one third of predicted genes over 500 bp in length having no significant hit in the databases. No genomic synteny was found between SJ and its closest whole genome sequenced relative at that time, *Bdellovibrio bacteriovorus* HD100.

**Figure 3:**
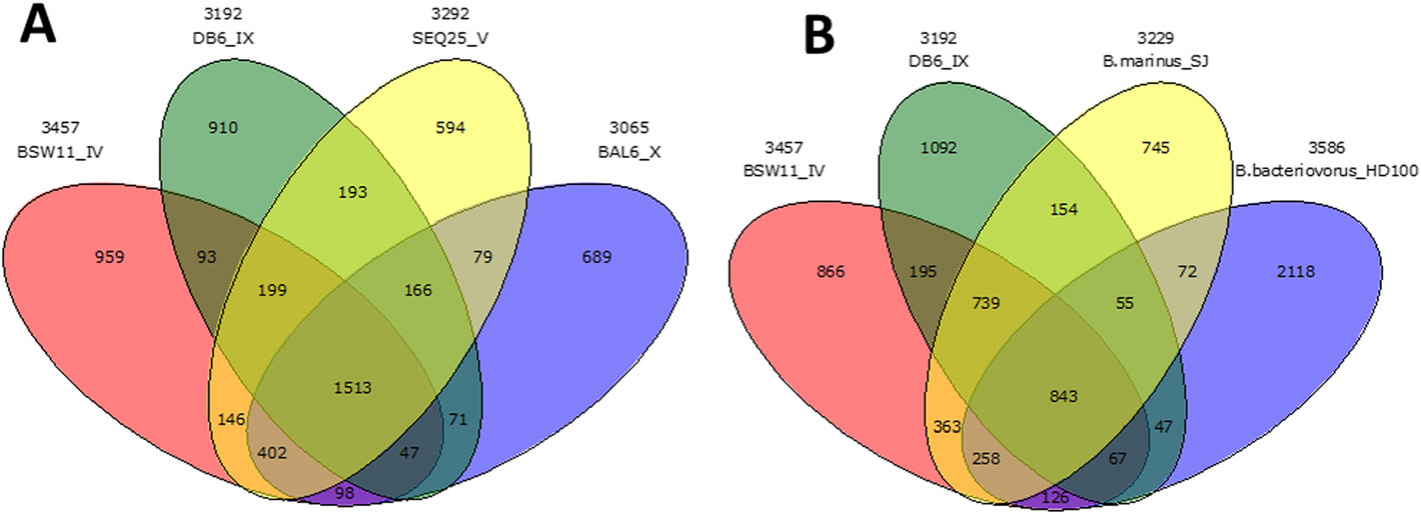
**Venn diagram of shared and unique genes in (A) the four newly sequenced Bacteriovorax isolates, (B) three *Bacteriovorax *strains and *Bdellovibrio bacteriovorus *HD100.** Orthology was assumed using the best reciprocal BLASTP matches (cutoff P value = 10–9).

We found that even within the genus *Bacteriovorax*, the genomic sequences were highly divergent with an average identity of 70%. A Venn diagram summarizing the comparison of the four *Bacteriovorax* isolates is presented in Figure 3. As shown in the diagram, a core of 1,513 proteins is shared by all four *Bacteriovorax* genomes and each encodes many proteins without orthologs in the other three (Figure 3A). When compared to the freshwater/terrestrial *Bdellovibrio bacteriovorus* HD 100, only a total of 843 genes were shared between all BALO members (Figure 3B). The calculated ANI [41] for BALO members (Additional file 2: Table S2) is below 75%, which is the threshold for the scores to be reliable. The AAI among the five *Bacteriovorax* genomes ranged between 50% to 60% (Additional file 3: Table S3), also significantly lower than the typical values found for species within a genus (73%-99.5%) [42,43]. Currently, several proposals to clarify and revise the systematics of BALOs are under consideration.

### Comparisons of BALOs and non-predatory bacteria

Phylogenetically, most genera of BALOs (including *Bacteriovorax*) are classified as *Deltaproteobacteria*. Members of this class are found in diverse environments with various lifestyles such as *Myxococcus xanthus* which is characterized by its gliding motility and wolf pack predatory strategy to prey on other bacteria [44], *Pelobacter carbinolicus* which grows by using iron and sulfur as electron acceptors [45], and the focus of this study, the obligate predators *Bacteriovorax* spp. which replicate within the periplasmic space of prey bacteria. Although their ecological features are distinct, the genomes of *Deltaproteobacteria* were found to exhibit some common characteristics. For example, most *Deltaproteobacteria*, including the *Bdellovibrio bacteriovorus* HD 100, typically possess two giant S1 ribosomal protein genes and high numbers of TonB receptors and ferric siderophore receptors which facilitate metal uptake and removal [46]. In contrast, only one giant S1 protein was found in the *Bacteriovorax marinus* SJ genome [40], and our study confirmed that this is the case for the other four *Bacteriovorax* genomes. *Bacteriovorax* genomes also encodes multiple TonB receptor proteins (6–11 copies) and ferric siderophore receptors (2–4 copies) that they may use for predation.

Using a reciprocal best match analysis with e-value cutoff of 10–9, 843 core genes were found to contain orthologs in all six BALO genomes including previously sequenced SJ and HD 100 genomes (see center of Figure 3B). Fifty nine of these genes (Additional file 4: Table S4) have no homologs with an E-value of 10–9 or lower to proteins from any non-predatory bacterium in the NCBI “nr” database. These genes, including periplasmic proteins, a radical activating enzyme and an outer membrane channel protein, may represent a core set of unique genes involved in the predatory process and prey interactions such as locating the prey, degradation and consumption of prey cellular content, formation of bdelloplast, synchronous nonbinary septation or release of progeny from the ghost cell.

## Conclusion

The genomes of four *Bacteriovorax* phylogenetic clusters isolated from the environment were sequenced. The genome sizes of the four strains were comparable with *Bacteriovorax* SJ but were slightly smaller than the two freshwater BALOs, *Bdellovibrio bacteriovorus* Tiberius and *B. bacteriovorus* HD100. Fifty-nine genes were identified that are conserved among BALOs, but not present in other organisms, that may be responsible for their predatory life style. The unique genomic features of *Bacteriovorax* that are essential for their ecological function were also reported.

## Abbreviations

BALOs: *Bdellovibrio* and Like Organisms; ANI: Average nucleotide identity; AAI: Average amino acid identity.

## Competing interests

The authors declare that they have no competing interests.

## Authors' contributions

JHB and HNW initiated and supervised the study. HC draft the manuscript, conducted wetlab work and performed electron microscopy. HC, LMB, DSL, TLD and NG annotated the genome. HC, PM, LMB and JHB worked on genome sequencing and assembly. HC, NL, JHB, PM and HNW discussed, analyzed the data and revised the manuscript. All authors read and approved the final manuscript.

## Supplementary Material

Additional file 1: Table S1 Associated MIGS record. Click here for file

Additional file 2: Table S2 Comparison of the average nucleotide identity (ANI) for the BALO genomes. ANI was calculated using ANI.pl script (https://github.com/chjp/ANI/blob/master/ANI.pl). All values are in percentages. Click here for file

Additional file 3: Table S3 Percentage of average amino acid identity (AAI) between BALO genomes. AAI calculation of all two-way BLAST conserved genes was computed using AAI.rb script (http://enveomics.blogspot.com/2013/10/aairb.html). Click here for file

Additional file 4: Table S4 Annotation of genes that are present in all BALO members but have no homologs from any non-predatory bacterium in NCBI’s “nr” database (E-value <10^-9^). Genes are listed by the protein number in BALO genomes. Click here for file
