# Supplementary material for: Draft genome sequences for the obligate bacterial predators Bacteriovorax spp. of four phylogenetic clusters
Source: Stand Genomic Sci. 2015 Mar 24;10:11. doi: 10.1186/1944-3277-10-11 (PMC4511183; doi:10.1186/1944-3277-10-11)
Supplement: Additional file 1: Table S1 — Associated MIGS record. [file 1944-3277-10-11-S1.doc]

*Additional file 1:* ***Table S1.*** *Associated MIGS record.*

| **MIGS-ID** | field name | description |
| --- | --- | --- |
| **MIGS-1** | Submit to INSDC/Trace archives |  |
| **1.1** | PID |  |
| **1.2** | Trace Archive |  |
| **MIGS-2** | MIGS CHECK LIST TYPE |  |
| **MIGS-3** | Project Name | [*Bacteriovorax*](http://dx.doi.org/10.1601/nm.3683) *spp.* |
| **MIGS-4** | Geographic Location | Breton Sound, LA (BSW11_IV);  Barataria Bay, LA (SEQ25_V);  Apalachicola Bay, FL (DB6_IX, BAL6_X) |
| **4.1** | Latitude | 29.63 -89.66 (BSW11_IV);  29.38 -89.98 (SEQ25_V);  29.67 -85.09 (DB6_IX, BAL6_X) |
| **4.2** | Longitude |
| **4.3** | Depth | not reported (BSW11_IV, SEQ25_V);  1.74m (DB6_IX, BAL6_X) |
| **4.4** | Altitude | not reported |
| **MIGS-5** | Time of Sample collection | April, 2011 (BSW11_IV);  June, 2011 (SEQ25_V); |
| **MIGS-6** | Habitat (EnvO) | Marine, estuarine |
| **6.1** | temperature | 15-30 °C |
| **6.2** | pH |  |
| **6.3** | Salinity | >0.5% |
| **6.4** | chlorophyll |  |
| **6.5** | conductivity |  |
|
| **6.6** | light intensity |  |
| **6.7** | dissolved organic carbon (DOC) |  |
| **6.8** | Current |  |
| **6.9** | atmospheric data |  |
| **6.10** | Density |  |
| **6.11** | alkalinity |  |
| **6.12** | dissolved oxygen |  |
| **6.13** | particulate organic carbon (POC) |  |
| **6.14** | phosphate |  |
| **6.15** | Nitrate |  |
| **6.16** | Sulfates |  |
| **6.17** | Sulfides |  |
| **6.18** | primary production |  |
| **MIGS-7** | Subspecific genetic lineage |  |
| **MIGS-9** | Number of replicons |  |
| **MIGS-10** | Extrachromosomal elements |  |
| **MIGS-11** | Estimated Size |  |
| **MIGS-12** | Reference for biomaterial or Genome report |  |
| **MIGS-13** | Source material identifiers |  |
| **MIGS-14** | Known Pathogenicity | None |
|
| **MIGS-15** | Biotic Relationship | Free living/ parasitic |
| **MIGS-16** | Specific Host |  |
| **MIGS-17** | Host specificity or range (taxid) |  |
| **MIGS-18** | Health status of Host |  |
| **MIGS-19** | Trophic Level |  |
| **MIGS-22** | Relationship to Oxygen | Aerobic |
| **MIGS-23** | Isolation and Growth conditions |  |
| **MIGS-27** | Nucleic acid preparation |  |
| **MIGS-28** | Library construction | 3KB 454 PE. insert Illumina fragment |
| **28.1** | Library size | 327bp avg |
| **28.2** | Number of reads |  |
| **28.3** | vector |  |
| **MIGS-29** | Sequencing method | Illumina GAii, 454 GS FLX Titanium |
| **MIGS-30** | Assembly | Newbler 2.6, CLC 5.0, CA7.0 |
| **30.1** | Assembly method |  |
| **30.2** | estimated error rate |  |
| **30.3** | method of calculation |  |
| **MIGS-31** | Finishing strategy | Improved-high-quality draft |
| **31.1** | Status |  |
| **31.2** | coverage | 81 X -700X hybrid coverage |
| **31.3** | contigs |  |
| **MIGS-32** | Relevant SOPs |  |
| **MIGS-33** | Relevant e-resources |  |
