# Supplementary material for: Draft genome sequences for the obligate bacterial predators Bacteriovorax spp. of four phylogenetic clusters
Source: Stand Genomic Sci. 2015 Mar 24;10:11. doi: 10.1186/1944-3277-10-11 (PMC4511183; doi:10.1186/1944-3277-10-11)
Supplement: Additional file 2: Table S2 — Comparison of the average nucleotide identity (ANI) for the BALO genomes. ANI was calculated using ANI.pl script (https://github.com/chjp/ANI/blob/master/ANI.pl). All values are in percentages. [file 1944-3277-10-11-S2.docx]

Additional file 2: **Table S2.** Comparison of the average nucleotide identity (ANI) for the BALO genomes. ANI was calculated using ANI.pl script (<https://github.com/chjp/ANI/blob/master/ANI.pl>). All values are in percentages.

|  | ***Bx. sp.* BSW11_IV** | ***Bx. sp.* SEQ25_V** | ***Bx. sp.***  **DB6_IX** | ***Bx. sp.***  **BAL6_X** | ***Bx. marinus***  **SJ** | ***B. bacteriovorus***  **HD100** |
| --- | --- | --- | --- | --- | --- | --- |
| ***Bx. sp.* BSW11_IV** | ̶ | 67.7 | 67.8 | 67.54 | 67.13 | 61.42 |
| ***Bx. sp.* SEQ25_V** | 67.91 | ̶ | 68.9 | 68.17 | 67.35 | 61.9 |
| ***Bx. sp.* DB6_IX** | 67.8 | 68.89 | ̶ | 68.72 | 67.58 | 61.11 |
| ***Bx. sp.* BAL6_X** | 67.42 | 68.19 | 68.73 | ̶ | 67.23 | 61.77 |
| ***Bx. marinus* SJ** | 67.25 | 67.37 | 67.52 | 67.03 | ̶ | 61.85 |
| ***B. bacteriovorus*HD100** | 61.7 | 62.17 | 61.09 | 61.53 | 62.32 | ̶ |
