# Supplementary material for: Draft genome sequences for the obligate bacterial predators Bacteriovorax spp. of four phylogenetic clusters
Source: Stand Genomic Sci. 2015 Mar 24;10:11. doi: 10.1186/1944-3277-10-11 (PMC4511183; doi:10.1186/1944-3277-10-11)
Supplement: Additional file 3: Table S3 — Percentage of average amino acid identity (AAI) between BALO genomes. AAI calculation of all two-way BLAST conserved genes was computed using AAI.rb script (http://enveomics.blogspot.com/2013/10/aairb.html). [file 1944-3277-10-11-S3.docx]

Additional file 3: **Table S3.** Percentage of average amino acid identity (AAI) between BALO genomes. AAI calculation of all two-way BLAST conserved genes was computed using AAI.rb script (http://enveomics.blogspot.com/2013/10/aairb.html).

|  | ***Bx. sp.* BSW11_IV** | ***Bx. sp.* SEQ25_V** | ***Bx. sp.***  **DB6_IX** | ***Bx. sp.***  **BAL6_X** | ***Bx. marinus***  **SJ** | ***B. bacteriovorus***  **HD100** |
| --- | --- | --- | --- | --- | --- | --- |
| ***Bx. sp.* BSW11_IV** | ̶ | 52.6 | 52.21 | 50.93 | 53.46 | 38.23 |
| ***Bx. sp.* SEQ25_V** | 52.55 | ̶ | 59.25 | 55.72 | 51.76 | 37.72 |
| ***Bx. sp.* DB6_IX** | 52.15 | 59.25 | ̶ | 55.84 | 52 | 37.67 |
| ***Bx. sp.* BAL6_X** | 50.95 | 55.67 | 55.8 | ̶ | 51.42 | 37.6 |
| ***Bx. marinus* SJ** | 53.52 | 51.78 | 52.05 | 51.44 | ̶ | 38.15 |
| ***B. bacteriovorus*HD100** | 38.13 | 37.83 | 37.7 | 37.68 | 38.19 | ̶ |
