# Supplementary material for: Draft genome sequences for the obligate bacterial predators Bacteriovorax spp. of four phylogenetic clusters
Source: Stand Genomic Sci. 2015 Mar 24;10:11. doi: 10.1186/1944-3277-10-11 (PMC4511183; doi:10.1186/1944-3277-10-11)
Supplement: Additional file 4: Table S4 — Annotation of genes that are present in all BALO members but have no homologs from any non-predatory bacterium in NCBI’s “nr” database (E-value <10-9). Genes are listed by the protein number in BALO genomes. [file 1944-3277-10-11-S4.docx]

Additional file 4: **Table S4.** Annotation of genes that are present in all BALO members but have no homologs from any non-predatory bacterium in NCBI’s “nr” database (E-value <10^-9^). Genes are listed by the protein number in BALO genomes.

| **Annotation** | ***B. bacteriovorus***  **HD100** | ***Bx. marinus***  **SJ** | ***Bx. sp.* BSW11_IV** | ***Bx. sp.* SEQ25_V** | ***Bx. sp.***  **DB6_IX** | ***Bx. sp.***  **BAL6_X** |
| --- | --- | --- | --- | --- | --- | --- |
| radical activating enzyme | Bd0040 | BMS_2203 | M899_2143 | M900_1223 | M901_2823 | M902_3251 |
| pilQ; putative pilus assembly transmembrane protein; K02280 pilus assembly protein CpaC | Bd0112 | BMS_0179 | M899_0182 | M900_0243 | M901_0358 | M902_1315 |
| hypothetical protein | Bd0148 | BMS_2896 | M899_1253 | M900_2297 | M901_0860 | M902_0514 |
| hypothetical protein | Bd0176 | BMS_1978 | M899_0306 | M900_2338 | M901_1938 | M902_0965 |
| TonB-like protein; K03832 periplasmic protein TonB | Bd0180 | BMS_2733 | M899_2759 | M900_2104 | M901_1670 | M902_2081 |
| hemagglutinin/hemolysin-like protein | Bd0209 | BMS_1619 | M899_3037 | M900_2084 | M901_2837 | M902_2873 |
| hypothetical protein | Bd0256 | BMS_2829 | M899_2428 | M900_2436 | M901_0518 | M902_3033 |
| rhs; rhs family protein | Bd0328 | BMS_1273 | M899_2183 | M900_1653 | M901_0551 | M902_2788 |
| hypothetical protein | Bd0416 | BMS_0267 | M899_1502 | M900_0335 | M901_2299 | M902_0272 |
| hypothetical protein | Bd0427 | BMS_2972 | M899_1325 | M900_0417 | M901_2388 | M902_1925 |
| hypothetical protein | Bd0474 | BMS_3225 | M899_3310 | M900_2626 | M901_1092 | M902_1710 |
| hypothetical protein | Bd0485 | BMS_2292 | M899_2394 | M900_1817 | M901_1248 | M902_1115 |
| putative lipopolysaccharide heptosyltransferase-I | Bd0616 | BMS_0090 | M899_0101 | M900_0165 | M901_1681 | M902_1391 |
| putative protease (EC:3.4.24.-); K01417 [EC:3.4.24.-] | Bd0751 | BMS_2181 | M899_0344 | M900_1178 | M901_1882 | M902_0287 |
| putative protease | Bd0754 | BMS_2178 | M899_0341 | M900_1175 | M901_1886 | M902_0284 |
| hypothetical protein | Bd0760 | BMS_0653 | M899_1190 | M900_0761 | M901_1733 | M902_2009 |
| hypothetical protein | Bd0782 | BMS_2797 | M899_1863 | M900_1696 | M901_2640 | M902_0575 |
| hypothetical protein | Bd0803 | BMS_0488 | M899_3048 | M900_0618 | M901_2473 | M902_0355 |
| hypothetical protein | Bd0857 | BMS_2958 | M899_1310 | M900_0431 | M901_1286 | M902_1940 |
| tolC; outer membrane export factor; K12340 outer membrane channel protein TolC | Bd0887 | BMS_0452 | M899_0047 | M900_1458 | M901_0278 | M902_0766 |
| hypothetical protein | Bd1129 | BMS_1726 | M899_3065 | M900_0624 | M901_2479 | M902_3054 |
| ybaN; hypothetical protein; K09790 hypothetical protein | Bd1144 | BMS_2427 | M899_0756 | M900_1914 | M901_1786 | M902_2306 |
| microtubule binding protein | Bd1167 | BMS_2568 | M899_3054 | M900_A0164 | M901_0957 | M902_2540 |
| hypothetical protein | Bd1176 | BMS_2372 | M899_0714 | M900_1870 | M901_0129 | M902_2697 |
| hypothetical protein | Bd1291 | BMS_0123 | M899_0130 | M900_0192 | M901_2737 | M902_1363 |
| hypothetical protein | Bd1312 | BMS_1218 | M899_1663 | M900_1264 | M901_2766 | M902_0867 |
| hypothetical protein | Bd1377 | BMS_2494 | M899_0296 | M900_A0155 | M901_1767 | M902_2533 |
| hypothetical protein | Bd1501 | BMS_0585 | M899_2961 | M900_0702 | M901_3159 | M902_0447 |
| general secretory pathway protein K; K02460 general secretion pathway protein K | Bd1587 | BMS_1263 | M899_2194 | M900_1663 | M901_2165 | M902_2798 |
| putative organic solvent tolerance protein | Bd1765 | BMS_2011 | M899_2091 | M900_1584 | M901_2094 | M902_3207 |
| hypothetical protein | Bd1785 | BMS_3371 | M899_0970 | M900_0822 | M901_0069 | M902_0908 |
| hypothetical protein | Bd1797 | BMS_3162 | M899_3258 | M900_2535 | M901_2503 | M902_1753 |
| hypothetical protein | Bd1818 | BMS_2171 | M899_0334 | M900_1168 | M901_1891 | M902_1130 |
| hypothetical protein | Bd1924 | BMS_1548 | M899_1966 | M900_A0334 | M901_2536 | M902_3135 |
| hypothetical protein | Bd1946 | BMS_2012 | M899_2092 | M900_1585 | M901_2095 | M902_3208 |
| hypothetical protein | Bd1991 | BMS_1256 | M899_1079 | M900_A0054 | M901_1382 | M902_3071 |
| spb1 gene forserine protease (AJ428902) related protein | Bd2274 | BMS_0677 | M899_2413 | M900_2657 | M901_2919 | M902_1950 |
| hypothetical protein | Bd2365 | BMS_1130 | M899_0583 | M900_1468 | M901_2666 | M902_2327 |
| hypothetical protein | Bd2429 | BMS_1410 | M899_0271 | M900_A0130 | M901_1651 | M902_3157 |
| hypothetical protein | Bd2555 | BMS_2162 | M899_2087 | M900_1579 | M901_2089 | M902_2757 |
| hypothetical protein | Bd2642 | BMS_1227 | M899_2235 | M900_1704 | M901_0711 | M902_1211 |
| hypothetical protein | Bd2659 | BMS_1732 | M899_2523 | M900_0251 | M901_0808 | M902_2399 |
| hypothetical protein | Bd2684 | BMS_0615 | M899_2937 | M900_0723 | M901_0389 | M902_0629 |
| hypothetical protein | Bd2744 | BMS_1725 | M899_1775 | M900_A0073 | M901_1531 | M902_0348 |
| hypothetical protein | Bd2754 | BMS_1977 | M899_0305 | M900_2816 | M901_0946 | M902_1534 |
| hypothetical protein | Bd2832 | BMS_2622 | M899_0842 | M900_A0195 | M901_3113 | M902_0802 |
| hypothetical protein | Bd2926 | BMS_2180 | M899_0343 | M900_1177 | M901_1883 | M902_0285 |
| hypothetical protein | Bd2929 | BMS_2177 | M899_0340 | M900_1174 | M901_1887 | M902_0283 |
| hypothetical protein | Bd3133 | BMS_1854 | M899_1581 | M900_A0304 | M901_1227 | M902_3291 |
| hypothetical protein | Bd3143 | BMS_3180 | M899_3272 | M900_2562 | M901_1054 | M902_1741 |
| hypothetical protein | Bd3348 | BMS_3409 | M899_3483 | M900_2796 | M901_1770 | M902_1553 |
| hypothetical protein (EC:3.6.3.14) | Bd3355 | BMS_2575 | M899_2559 | M900_A0344 | M901_2523 | M902_0330 |
| D-alanyl-D-alanine carboxypeptidase (EC:3.4.16.4) | Bd3459 | BMS_2102 | M899_2036 | M900_1537 | M901_0930 | M902_3188 |
| hypothetical protein (EC:3.4.21.-); K01362 [EC:3.4.21.-] | Bd3531 | BMS_2431 | M899_0757 | M900_1915 | M901_1784 | M902_2304 |
| hypothetical protein | Bd3603 | BMS_2808 | M899_2857 | M900_2200 | M901_0672 | M902_0620 |
| hypothetical protein | Bd3604 | BMS_2807 | M899_2856 | M900_2199 | M901_0673 | M902_0621 |
| hypothetical protein | Bd3743 | BMS_1865 | M899_1572 | M900_A0293 | M901_1022 | M902_3303 |
| hypothetical protein | Bd3748 | BMS_0407 | M899_0307 | M900_0585 | M901_2543 | M902_2336 |
| putative arsenical pump-driving ATPase (EC:3.6.3.16) | Bd3763 | BMS_3008 | M899_3113 | M900_0573 | M901_2255 | M902_1879 |
